# Incidence and Associated Factors of SARS-CoV-2 Infection Post-mRNA-1273 Booster Vaccination in Health-Care Workers

**DOI:** 10.3390/vaccines11020481

**Published:** 2023-02-19

**Authors:** Anshari Saifuddin Hasibuan, Sukamto Koesnoe, Alvina Widhani, Muhadi Muhadi, Hamzah Shatri, Eka Ginanjar, Evy Yunihastuti, Pradana Soewondo, Sally Aman Nasution, Samsuridjal Djauzi, Lies Dina Liastuti, Trimartani Koento, Sumariyono Sumariyono, Astri Mulyantini

**Affiliations:** 1 Division of Allergy and Clinical Immunology, Department of Internal Medicine, Faculty of Medicine, University of Indonesia/Cipto Mangunkusumo General Hospital, Jakarta 10430, Indonesia; 2 Division of Cardiology, Department of Internal Medicine, Faculty of Medicine, University of Indonesia/Cipto Mangunkusumo General Hospital, Jakarta 10430, Indonesia; 3 Division of Psychosomatic and Palliative Care, Department of Internal Medicine, Faculty of Medicine, University of Indonesia/Cipto Mangunkusumo General Hospital, Jakarta 10430, Indonesia; 4 Division of Endocrine System, Metabolism and Diabetes, Department of Internal Medicine, Faculty of Medicine, University of Indonesia/Cipto Mangunkusumo General Hospital, Jakarta 10430, Indonesia; 5 Indonesian Society of Internal Medicine, Jakarta 10430, Indonesia; 6 Adult Immunization Task Force, Indonesian Society of Internal Medicine, Jakarta 10430, Indonesia; 7 Cipto Mangunkusumo General Hospital, Jakarta 10430, Indonesia

**Keywords:** vaccination, COVID-19, health-care workers

## Abstract

The COVID-19 pandemic has caused significant morbidity and mortality worldwide, especially among health-care workers. One of the most important preventive measures is vaccination. This study examined factors associated with the incidence rate of SARS-CoV-2 infection after mRNA-1273 booster vaccination (preceded by the CoronaVac primary vaccination) and the antibody profile of health-care workers at one of the tertiary hospitals in Indonesia. This was a combined retrospective cohort and cross-sectional study. Three hundred health-care workers who were given the mRNA-1273 booster vaccine a minimum of 5 months prior to this study were randomly selected. Participants were then interviewed about their history of COVID-19 vaccination, history of SARS-CoV-2 infection, and comorbidities. Blood samples were taken to assess IgG sRBD antibody levels. The median antibody level was found to be 659 BAU/mL (min 37 BAU/mL, max 5680 BAU/mL, QIR 822 BAU/mL) after the booster, and this was not related to age, sex, comorbidities, or adverse events following immunization (AEFI) after the booster. SARS-CoV-2 infection after the booster was correlated with higher antibody levels. In sum, 56 participants (18.6%) experienced SARS-CoV-2 infection after the mRNA-1273 booster vaccination within 5 months. Incidence per person per month was 3.2%. Age, sex, diabetes mellitus type 2, hypertension, obesity, and post-booster AEFI were not related to COVID-19 incidence after the booster. History of SARS-CoV-2 infection before the booster vaccination was significantly associated with a reduced risk of SARS-CoV-2 infection after booster vaccination, with a relative risk (RR) of 0.21 (95% CI 0.09–0.45, *p* < 0.001).

## 1. Introduction

Coronavirus disease 2019 (COVID-19) was first reported in December 2019 in China and was later identified as severe acute respiratory syndrome coronavirus 2 (SARS-CoV-2). This is a disease that causes pneumonia in infected individuals. A pandemic was declared by the World Health Organization in March 2020 [1]. By 2 January 2023, COVID-19 cases had reached more than 660 million with 6.6 million deaths worldwide. Case numbers continue to grow with the emergence of new variants, such as omicron. In Indonesia on the same day, COVID-19 cases had reached more than 6.7 million with 161 thousand deaths [2]. According to the WHO Strategic Advisory Group of Experts on Immunization (SAGE) Roadmap, the priority population for vaccination is health-care workers, who are at high risk of infection, transmission, and death from SARS-CoV-2, because they cannot effectively maintain distance while doing their jobs [3]. When the second wave, dominated by the delta variant, appeared, there was concern from Indonesian health-care workers who were at the forefront of COVID-19 handling due to the large number of deaths, even though they had been fully vaccinated with the CoronaVac vaccine (produced by Sinovac, China). Nearly 100% of health-care workers had already been vaccinated when the delta wave appeared in Indonesia. However, their death rate continued to increase. The number of doctor deaths reached 216 in July 2021 alone, the highest monthly rate during the pandemic [4,5]. The Indonesian government decided to administer a booster vaccination using the mRNA-1273 vaccine (produced by Moderna, USA) to health-care workers in order to reduce the risk of death.

Several factors that are associated with SARS-CoV-2 infection include age, sex, comorbidities such as diabetes mellitus, hypertension, and obesity, and history of COVID-19. Meister et al. reported that the >60-year age-group had a lower chance of being infected than young adults, even though the risk of severe infection was greater [6]. Individuals with comorbidities also have a higher risk of being infected than healthy individuals. This can be seen from a Villar et al. study and others, which have reported higher risk of infection in groups with comorbidities such as diabetes mellitus, hypertension, and obesity. Being female has also been indicated as a higher risk factor of SARS-CoV-2 infection, although results of existing studies have come to different conclusion, several supporting studies were carried out by Porru et al. and Villar et al [7,8]. History of COVID-19 is known to reduce the risk of reinfection. This was supported by the studies of Hall et al. and Dhumal et al. [9,10].

Several factors are known to cause lower IgG antibody titers after COVID-19 vaccination (after primary vaccination with BNT162b2 produced by Pfizer/BioNTech), including age, sex, and comorbidities such as diabetes mellitus, obesity and hypertension [11]. Other studies have found a relationship between higher antibody levels and adverse events after immunization (AEFI) [12]. Studies on the antibody profiles of Indonesian health-care workers vaccinated with the mRNA-1273 booster vaccine are still limited. A literature search conducted by the authors found three studies assessing sRBD IgG antibody levels: Hidayat et al., Cucuwaningsih et al., and Sinto et al. Increased antibody levels were reported after being vaccinated with the mRNA booster vaccine in these studies. However, antibody levels after 5 months since booster vaccination and COVID-19 incidence were not reported in these studies [13,14,15]. Menni et al. reported that the effectiveness of the COVID-19 vaccine decreased after 5 months of COVID-19 vaccination [16].

Studies on COVID-19 booster vaccines, especially the mRNA-1273 platform, which was preceded by inactivated vaccines such as CoronaVac, are still very limited. This study is expected to increase knowledge about the effectiveness of the mRNA-1273 booster vaccine preceded by CoronaVac and factors associated with effectiveness, especially in health-care workers.

## 2. Method

### 2.1. Study Design

This study used a combined retrospective cohort and cross-sectional design. The retrospective cohort design was used to investigate COVID-19 incidence post-booster vaccination and the cross-sectional design to determine the relationship between antibody titer and age, sex, comorbidities (diabetes mellitus type 2, hypertension, obesity), history of COVID-19, and AEFI after booster vaccination. The main outcome was confirmed COVID-19 cases after booster vaccination and the secondary outcome was the antibody profile after the booster. Participants were health-care workers who worked at Cipto Mangunkusumo General Hospital (RSCM), Jakarta. The inclusion criteria were age 18–59 years, working as a health-care worker, and having received the COVID-19 booster vaccination with the mRNA-1273 platform within the last 5 months, preceded by two primary CoronaVac vaccinations. The cutoff of 5 months was chosen in consideration of the fact that effectiveness and antibodies start to decrease 5 months after the vaccine is administered. In order to minimize outcome bias, the participants recruited were health-care workers who had direct daily contact with out- or inpatients, doctors, nurses, and midwives. Exclusion criteria included the participant being pregnant or breastfeeding, having coronary heart disease or an autoimmune disease, undergoing hemodialysis, having hematological and non-hematological malignancies, HIV infection, undergoing organ transplant procedures, currently being treated with immunosuppressants, incomplete screening data, and not working or studying at RSCM. This study involved 300 participants from the total of 2114 health-care workers at RSCM who met the inclusion criteria and had received the mRNA-1273 booster vaccine. COVID-19 incidence was assessed from the number of COVID-19-positive confirmations based on the shortest time interval between interview and booster vaccination for all participants to prevent bias in the observation outcomes. Incidence of first recorded COVID-19-positive case after booster were assessed as incidence outcome. Second or third COVID-19-positive cases are presented.

sRBD IgG antibodies were examined using an Architect device with the two-step chemiluminescent microparticle immunoassay (CMIA) technique. Blood samples, reagent that consisted of paramagnetic microparticles coated with SARS-CoV-2 antigen, and diluent assays were combined and incubated. IgG antibodies against SARS-CoV-2 were present in the samples bound to the microparticles coated with the SARS-CoV-2 antigen. The mixture was then washed and acridinium-labeled human anti-IgG conjugate was added to form a reaction mixture, which was then incubated. After completion of washing cycles, pre-trigger and trigger solution were added. The result was reactive if antibody titer was ≥50 AU/mL and nonreactive if it was <50 AU/mL, with a measurement range of 21–40,000 AU/mL. Units were converted to BAU/mL in accordance with WHO criteria with a conversion rate of 0.142 from the results obtained according to the reagent used.

### 2.2. Data Collection and Statistical Analysis

**Figure 1:**
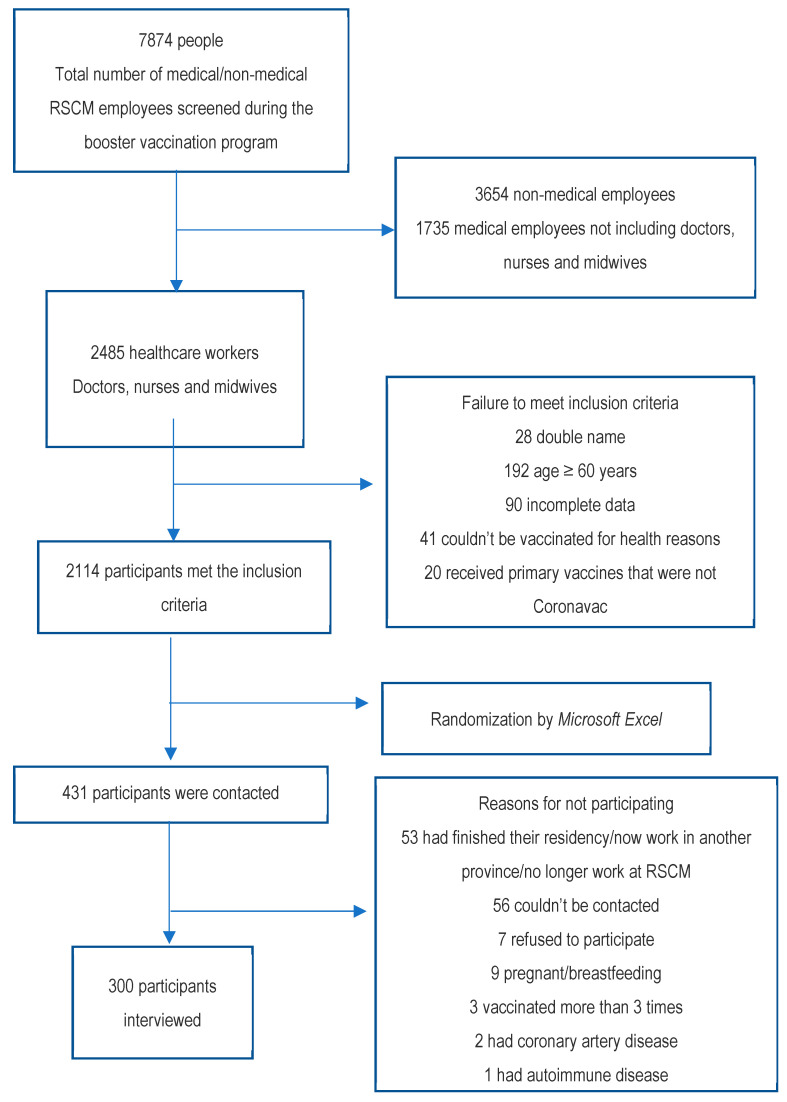
Flow of participant recruitment. In sum, 7874 RSCM employees with either medical or nonmedical backgrounds received the mRNA-1273 booster vaccination and had been recorded in the screening data of the COVID-19 booster program. From these 7874, 300 participants were obtained and interviewed.

Participants were selected based on screening data that had been collected from the RSCM COVID-19 vaccination team during the COVID-19 booster vaccination program (Figure 1). From the existing data, the participants were then selected randomly using a simple random sampling method according to the inclusion criteria. Then, further interviews were conducted and blood samples were taken to determine IgG sRBD antibody levels. Interviews were conducted for the purpose of collecting information regarding the variables, such as age, weight and height, sex, history of comorbidities (hypertension and type 2 diabetes mellitus), history of primary and booster vaccinations, and history of SARS-CoV-2 infection. Data were analyzed using Statistical Product and Service Solutions (SPSS) for Windows version 24.0. The characteristics of research participants are presented in the form of tables and narratives. Categorical data are presented in the form of frequency distribution. Numerical data are presented in the form of the mean and standard deviation or in the form of the median and interquartile range. Bivariate analysis of each independent variable was conducted using the chi-squared test or Fisher’s exact. The Mann–Whitney test was used to determine the correlation between variables and antibody levels.

## 3. Results

### 3.1. Participant Characteristics

The majority of participants in this study were under 50 years of age, totaling 270 participants (90%) and female, totaling 232 participants (77.3%). The number of participants with comorbidities were: diabetes mellitus type 2, 10 (3.3%); hypertension, 36 (12%); and obesity, 151 participants (50.3%). The majority of participants stated that they had been infected with SARS-CoV-2 once—176 (58.7%), 61 (20.3%) had never been infected with SARS-CoV-2, and 121 (40.3%) said that they had been infected with COVID-19 before the booster vaccination (Table 1).

### 3.2. Incidence and Variables Related to Infection after Booster

**Figure 2:**
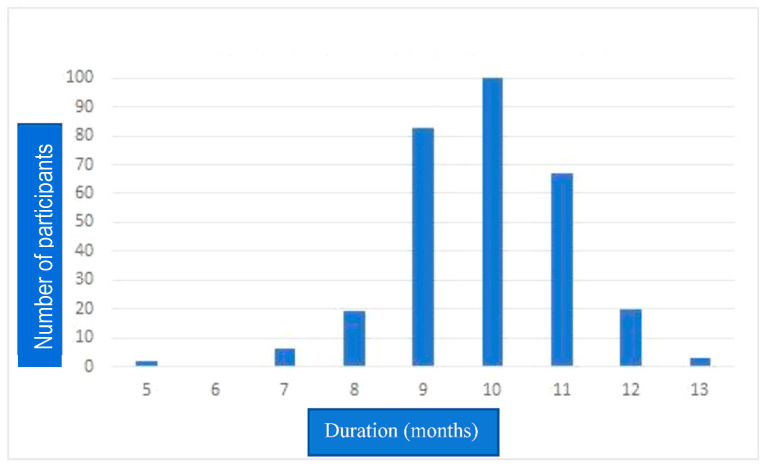
Interval between interview and booster vaccination. This figure shows variation in the time interval between booster vaccination and participants’ interview. The researchers set 5 months as the interval of outcome observation after booster vaccination for equivalence and to avoid bias.

The time interval between booster vaccination and participants’ interviews was different (Figure 2), varying from 5 months to 13 months. As a result, an equal time interval in observation was needed to prevent outcome bias. A 5-month interval was taken as the shortest time between interview and booster vaccination among all participants. A total of 56 participants had confirmed SARS-CoV-2 infection within 5 months, giving a cumulative incidence of 18.6%. The incidence per person per month was 3.2%. Confirmed COVID-19 cases increased with every month of the 5 months after booster vaccination (Table 2). Bivariate analysis was carried out to assess the relationship between the variables studied and post-booster infection using the chi-squared test. It was found that the variables of age, sex, hypertension, diabetes, obesity and post-booster AEFI had no association with the incidence of SARS-CoV-2 infection after booster vaccination (Table 3). The variable that was found to be associated with COVID-19 incidence post-booster vaccination was a history of COVID-19 before the booster, with a relative risk (RR) of 0.21 (95% CI 0.09–0.45, *p* < 0.001).

**Figure 3:**
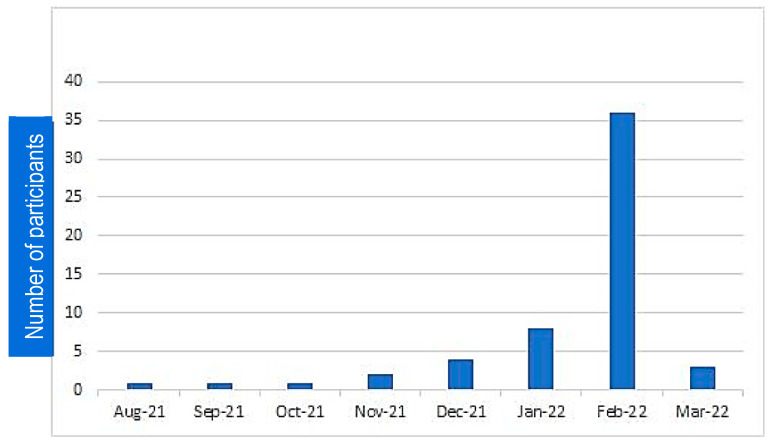
COVID-19 events within 5 months of the booster vaccination (n = 56). As seen in this figure, there was a substantial increase in COVID-19 cases among the 300 subjects between December 2021 and February 2022. This is in line with the third wave of COVID-19 in Indonesia.

**Figure 4:**
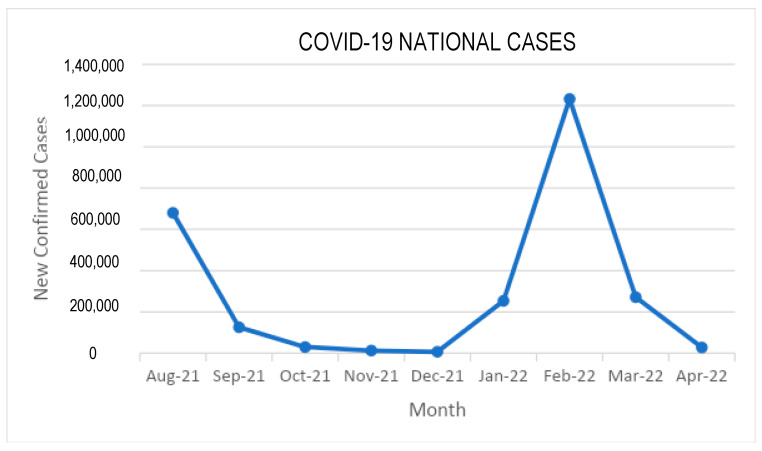
Confirmed COVID-19 cases in Indonesia (August 2021–April 2022) based on World Healh Organization and COVID-19 Response Acceleration Task Force Republic of Indonesia data. As seen in this figure, there was a substantial increase in COVID-19 cases from December 2021 to February 2022. Indonesia experienced a third wave of COVID-19 between these months, which was characterized by the omicron variant.

COVID-19 incidence began to increase in December 2021, peaked in February 2022, and then began to decrease again (Figure 3). This increase could be associated with the omicron variant that appeared in the third COVID-19 wave in Indonesia, which started in January 2022 and declined between mid-February and April 2022 [17] as seen in (Figure 4).

**Figure 5:**
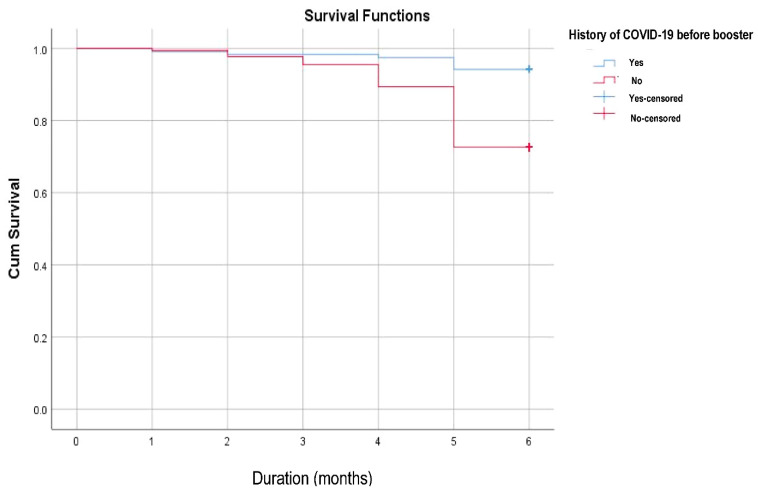
Kaplan–Meier analysis of COVID-19 incidence among participants with a history of COVID-19 and participants without a history of COVID-19 (n = 300). This figure displays the difference in confirmed SARS-CoV-2 infections after booster vaccination among subjects with history of COVID-19 before the booster (n = 121) and those without a history of COVID-19 before the booster (n = 179).

It can be seen that participants with a history of COVID-19 had a lower percentage of confirmed SARS-CoV-2 infection after booster compared to participants without a history of COVID-19. This was measured from the first month to the fifth month after booster vaccination, with a survival rate of 94.2 % vs. 72.6% (*p* < 0.001) (Figure 5).

**Figure 6:**
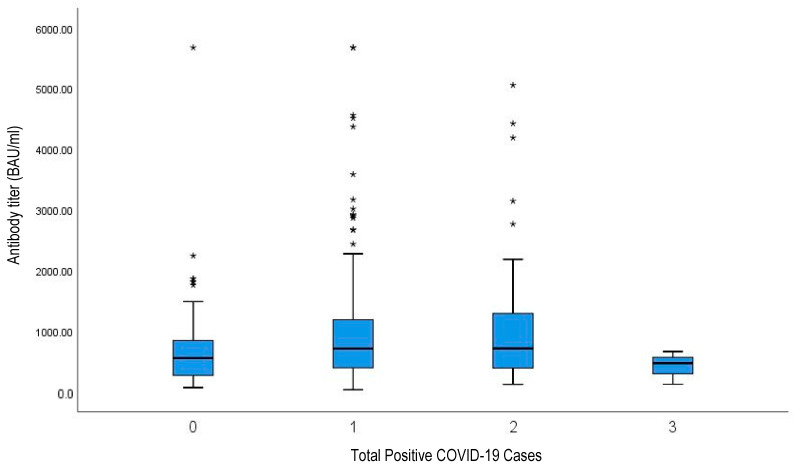
Distribution of antibody titer in correlation to total positive COVID-19 cases before and after booster. As seen in the figure, increased antibody levels were observed in participants who had confirmed COVID-19 cases 0, 1, or 2 times, but lower levels were observed in participants who had contracted COVID-19 3 times. (* outliers / antibody titer with extreme value).

The median sRBD antibody level of the 300 participants was 659 BAU/mL (min 37 BAU/mL, max 5680 BAU/mL, QIR 822 BAU/mL). The median duration of sRBD antibody testing after booster vaccination was 315 days (min 148 days, max 413 days). Median IgG sRBD antibody test results were analyzed against total confirmed COVID-19 cases, both before and after the booster vaccination (Figure 6). The median antibody level was 553 BAU/mL (IQR 628 BAU/mL) in participants that had never had a confirmed COVID-19 case, 710 BAU/mL (IQR 796 BAU/mL) in participants with one confirmed COVID-19 case, 712 BAU/mL (IQR 903 BAU/mL) in participants with two confirmed COVID-19 cases, and 417 BAU/mL (SD 274 BAU/mL) in participants with three confirmed COVID-19 cases. The median duration after COVID-19 booster vaccination until blood sampling was conducted was 330 days for participants who had never had a confirmed COVID-19 case, 312 days for participants who had one confirmed COVID-19 case, 306 days for participants who had two confirmed COVID-19 cases, and 268 days for participants with three confirmed COVID-19 cases.

The correlation between variables and antibody titer was analyzed using the Mann–Whitney test. It was found that age, sex, hypertension, diabetes, obesity and post-booster AEFI vaccination were not significantly related to antibody levels (Table 4). The variable that was found to have a significant correlation was infection after booster vaccination. Median antibody level in participants who were not infected after the booster compared to those who were infected was 551 BAU/mL vs. 1005 BAU/mL, with *p*-value < 0.001.

## 4. Discussion

In this study, 151 participants (50.3%) were obese (body mass index (BMI) ≥ 25). The percentage of health-care workers who were obese is quite high, but it is actually in line with several studies that have been conducted in other countries. A study conducted by Kunyahamu et al. in Malaysia found that of 4241 health-care workers, 33.1% were overweight and 21.1% obese following the WHO criteria for BMI. They also found that 7.5% of health-care workers had such comorbidities as diabetes mellitus, asthma, hypertension, and cardiovascular disease [18]. Meanwhile, in this present study, 3.3% of participants had diabetes mellitus and 12% had hypertension. Research on obesity in health-care workers has also been reported in England by Kyle et al. From this study’s 20,103 participants, the percentage of health-care workers who were obese (BMI ≥ 30) reached 25.1% in nurses and 14.4% in other health workers (doctors, physiotherapists, etc.) [19].

In this present study, 176 participants (58.7%) had been infected with SARS-CoV-2 only once, and 61 participants (20.3%) had never been infected with SARS-CoV-2. The proportion of participants who had been infected (one to three times) was 79.7%. This is quite large when compared to several other previously reported studies. Research by Soebandrio et al. reported that 7.9% of health-care workers tested positive for SARS-CoV-2 from 1201 specimens collected in Indonesia from March to May 2020 [20]. Meanwhile, a study in Iran reported 5.62% positive cases (273 of 4854 specimens) in data collected from March to May 2020 [21]. The large number of health-care workers with a history of SARS-CoV-2 infection in our study could be due to the long period in which infection was recorded. History of infection was recorded from the start of the COVID-19 pandemic in Indonesia in March 2020 until the interviews were conducted in July 2022.

A total of 56 participants (18.6%) were confirmed positive for COVID-19 within 5 months after booster vaccination. Incidence per person per month was 3.2%. In addition, there was a consistent increase in incidence per month from the first month to the fifth month. In comparison, the incidence of COVID-19 in health-care workers in Israel reported by Spitzer et al. was 0.3% with a median observation interval of 39 days [22]. Menni et al. in England reported a shorter duration in which reduced vaccine effectiveness (waning) was found against infection 5 months after the second vaccination [16]. Another study reported by the CDC found that the effectiveness of three doses of mRNA vaccination reduced from 96% at 2 months postvaccination to 76% 4 months postvaccination and thereafter during delta waves. Meanwhile, the effectiveness during omicron waves decreased from 71% at 2 months postvaccination to 54% at 5 months and thereafter [23].

The increasing incidence of infection from January to February 2022, as seen in Table 2, may be correlated with the omicron variant. The omicron wave in Indonesia began in January 2022 and peaked in February, before starting to decline. Variants of concern (VOC), such as delta and omicron, caused COVID-19 vaccine effectiveness to decrease. Andrew et al. reported significant reduction in effectiveness of two doses of primary vaccination against the omicron variant compared to delta variant. In fact, they reported the effectiveness of two primary doses of ChAdOx1 vaccine as having no effect at all on symptomatic infection caused by the omicron variant at 25 weeks postvaccination. Booster doses with two primary doses of ChAdOx1 were able to increase effectiveness against omicron variants, but this did decrease with time. Booster with BNT162b2 increased effectiveness, reaching 62.4% within 4 weeks postvaccination, but this decreased to 39.6% after 10 weeks or more. Meanwhile, boosters with mRNA-1273 increased effectiveness, reaching 70.1% within 4 weeks after vaccination, but decreased to 60.9% after 10 weeks or more [24].

Bivariate analysis was carried out to assess the relationship between the variables studied and post-booster infection. It was found that age, sex, hypertension, diabetes, obesity and AEFI had no relationship to the incidence of COVID-19 after booster. In this study, no significant correlation was found between ages <50 years and ≥50 years and incidence of COVID-19 infection. The RR value was 0.51 (95% CI 0.17–1.53, *p* = 0.3). This result could be due to the significantly fewer participants aged above 50 years than those under 50 years, because the average age of retirement for health-care workers at RSCM is over 60 years.

In a study by Igari et al., it was found that age was associated with post-booster infection with the BNT vaccine. In this study, participants aged 20–29 years had a significant difference in the incidence of infection: 2.9% for those who had received the booster vaccine and 13.6% for those who had not. From the results of their multivariate analysis, it was found that age 20–49 years was associated with a higher incidence of infection after booster vaccines (aOR 9.7, 95% CI = 1.3–71.2). This result may be correlated with the social factor of younger participants being more active than older ones [25]. RSCM took preventive measures to avoid COVID-19 transmission in accordance with WHO recommendations, and we assume that the same preventive measures were taken in Japan. Different results were reported in a Vivaldi et al. study in England, which found no correlation between age and the risk of breakthrough infection after BNT162b2 or mRNA-1273 booster vaccination. [26]. 

Sex was not found to have a significant relationship with post-booster COVID-19 events, with an RR value of 1.07 (95% CI 0.60–1.92, *p* = 0.945). This is in line with a study by Porru et al., who likewise found no significant correlation between sex and SARS-CoV-2 infection, even though the participants of that study had not been vaccinated [7]. Different results were reported by Meister et al. and Villar et al., who concluded that there was a significant relationship between sex and risk of infection with SARS-CoV-2 [6,8]. Comorbidities in the form of hypertension, type 2 diabetes mellitus, and obesity had no relationship to the incidence of COVID-19 in this study, with RR values of 1.22 (95% CI 0.63–2.37 *p* = 0.722), 0.53 (95% CI 0.08–3.44, *p* = 0.722) and 0.53 (95% CI 0.08–3.44, *p* = 0.694) respectively. In the study of Vivaldi et al., there was no relationship between BMI, either after a primary or a booster vaccine, and the risk of infection in health-care workers. This could be due to the fact that some health workers had experienced a more severe COVID-19 infection than the general population at the start of the pandemic. It could be the result of better antibody persistence compared to those who experienced previous infections that were only asymptomatic or mild [26]. Our study results are different from those of Villar et al. and Meister et al. which concluded that groups with comorbidities, such as diabetes, hypertension, and obesity, have a higher risk of experiencing SARS-CoV-2 infection than groups without comorbidities [6,8]. The insignificant result from our study could be due to the fewer participants with hypertension and diabetes than participants without comorbidities. AEFI had no relationship to the incidence of COVID-19 after booster in this study. This is in line with a study conducted by Takeuchi et al., which concluded that there were more adverse reactions after the second injection of the mRNA type COVID-19 vaccine than the first injection. However, the adverse reactions that occurred more frequently after the second injection were not related to the spike IgG antibody levels formed [27].

The variable that had a significant relationship with post-booster SARS-CoV-2 infection was a history of SARS-CoV-2 infection before booster. These results are in accordance with a study conducted by Dhumal et al. that examined the relationship between the incidence of SARS-CoV-2 reinfection in a group of health-care workers who had previously been infected. It was concluded that previous episodes of SARS-CoV-2 infection provided better protection against reinfection. COVID-19 vaccination also provided additional protection in this group [10]. Another study conducted by Wang et al. concluded that vaccination after recovery from natural SARS-CoV-2 infection or “hybrid immunity” can increase both potency and coverage of humoral immune response to SARS-CoV-2 [28]. Results obtained by Bates et al. showed that SARS-CoV-2 infection before or after vaccination caused a significant increase in neutralizing antibody responses compared to only two vaccinations, both in terms of quantity and quality. They observed that booster vaccination 8 months after two doses of primary vaccine provided a significant increase in neutralizing antibodies against the delta variant of up to 6–12 times. Bates et al.’s results are consistent with the increase in neutralizing antibodies up to 8.5–15.7 times in the group with a history of COVID-19 (hybrid immunity) before being vaccinated twice. In addition to the role of humoral response, the role of cellular response via T cells is also known to play an important role in the response to COVID-19 vaccination and infection [29].

Cases of infection after being vaccinated, as seen in this study where participants were found to be infected with SARS-CoV-2 post-booster, show that immunity to SARS-CoV-2 is only temporary or imperfect. This imperfect immunity can result from the emergence of new virus variants that can evade natural immunity, as well as several other factors that must be considered in assessing the effectiveness of immune response to reinfection by SARS-CoV-2. In addition to the quantity of neutralizing antibody levels decreasing over time, the quality of these antibodies must also be considered. Batsch et al. found that T-cell response and neutralization were only seen in participants who achieved sRBD antibody titers above threshold. Only individuals with high IgG titers had a strong response to the specific RBD, N, and S proteins of SARS-CoV-2. Meanwhile, limited humoral responses to the three proteins can be seen in individuals who have low sRBD antibody titers. This could be due to the relationship between antibody levels and their function. A certain level of antibodies is required to achieve adequate functioning of the humoral and cellular responses. From this concept, the higher a person’s IgG sRBD value, the higher the quantity of neutralizing antibodies [30].

On the other hand, reinfection can be caused by low quality of the neutralizing antibody even though the sRBD antibody value is high. This case is referred to as “avidity” of IgG against its target epitope—RBD. Avidity is generated during antibody maturation, and failure to reach IgG with high avidity results in a lack of protective immunity against SARS-CoV-2 infection. This can explain why people with good sRBD IgG antibody levels still get infected: maybe only some IgG have high avidity for sRBD [31]. From these two studies, it can be concluded that a certain threshold is needed for both number and function of neutralizing antibodies to completely prevent SARS-CoV-2 infection. Until now, there is no global consensus on the value of neutralizing antibodies that can fully protect a person from infection with SARS-CoV-2. This lack of consensus is perhaps due to the difficulty involved in obtaining a threshold that is consistently protective for everyone. However, current research suggests that postvaccination neutralizing antibodies, although not completely preventing SARS-CoV-2 infection, can reduce the incidence of severe symptoms and hospitalization due to COVID-19.

In addition to neutralizing antibodies, the role of cellular immunity, such as T cells, cannot be neglected. Existing studies prove that T-cell response is very important in protection against SARS-CoV-2 infection. Specific T-cell responses play a role in virus elimination, prevent infection without the need for seroconversion, produce a strong memory against future infections, and help recognize new variants that appear [32]. This role of cellular immunity can also explain why people can be infected even when their IgG sRBD antibody level is high: their cellular immunity function may not be good.

In the present study, it was found that the average antibody level in health-care workers who had been vaccinated with booster mRNA-1273 with CoronaVac primary vaccine had a median of 659 BAU/mL with a duration of up to 315 days. These results are in line with the study of Hidayat et al. that was conducted in a tertiary hospital in Indonesia on 49 health-care workers, of which 41 (83.7%) had antibody levels reaching > 1000 AU/mL 3 months after the booster vaccination [13]. According to the total number of COVID-19-positive results before and after booster, participants who had never experienced SARS-CoV-2 infection had antibody levels with a median of 553 BAU/mL. This was lower than those who had been infected one or two times, with medians of 710 BAU/mL and 712 BAU/mL, respectively. However, these values were still better than participants who had been infected three times, who had a median of 417 BAU/mL. Three participants had experienced SARS-CoV-2 infection three times. All of them were under 50 years old and had no comorbidities. The time of blood sampling for IgG sRBD antibody test with booster vaccinations or last infection did not much differ among participants who had been infected one or two times.

Factors that affect antibody formation response to antigen/infection in each person vary widely. The lower antibody values that were obtained in the three participants with three incidents of infection can be linked to genetic factors. Genetic influences on the host are related to immune response to SARS-CoV-2 infection. Genetic factors that are known to play a role include polymorphisms in ACE2 receptor, TMPRSS2 receptor, and HLA genetic variation. Several studies have found that two variants of ACE2 (K26R and 1468V) have lower binding affinity for protein S in SARS-CoV-2. Patients with diabetes, hypertension and chronic obstructive pulmonary disease have also been found to have higher expression of ACE2 than healthy individuals, which may explain why this group are more susceptible to severe symptoms of COVID-19. In addition, single-nucleotide polymorphisms (SNPs) are associated with increased TMPRSS2 expression and reduced MX1 expression, which plays a role in interferon formation. Individuals with SNPs are more susceptible to SARS-CoV-2 infection due to increased expression of TMPRSS2 on the cell surface and poor cellular response to the virus. Allele variations in HLA such as HLA-B*46:01 are known to be associated with more severe symptoms of COVID-19 in Asian populations [33]. Unfortunately, these factors were not analyzed in this study.

In this study, it was found that age had no significant relationship with antibody levels in the participants. This is consistent with studies conducted by Sinto et al. and Bates et al., where no correlation was found between antibody levels and age [15,29]. Similar results were also obtained by Richards et al., in which there was no significant difference in antibody level in participants aged 50 years and over 50 years [34]. Antibody level did not display a significant difference between the sexes either. Cucuwaningsih et al. studied the levels of sRBD antibodies in 90 health-care workers who were vaccinated twice with CoronaVac primary followed by mRNA-1273 booster vaccination. Their results showed that there was a significant increase in sRBD antibodies from a median of 41.7 AU/mL to 28,394 AU/mL [14]. However, this increase was not significantly different when correlated with age. The same results were obtained by Sinto et al. and Richards et al., who also found no significant difference in antibody titers associated with sex [15,34]. In the present study, there were no significant differences in antibody titers associated with hypertension or obesity. This is in line with the results of a study conducted by Choi et al. that found no significant difference in neutralizing antibody levels or S-IgG levels 6 months after the second dose of mRNA vaccination in healthy versus comorbid groups [35]. Pellini et al. likewise found no significant difference in antibody titers between hypertension and healthy groups [36]. In the study of Hidayat et al., it was also found that there was no relationship between antibody levels and the variables of sex, age, BMI or comorbidities [13].

In this study, there was a significant relationship between the antibody levels of participants who experienced COVID-19 after the booster vaccination compared to those who did not. This result is in line with several previous studies. Anichini et al. found that antibody titers after the first dose of vaccination in the group that had never been infected with SARS-CoV-2 were lower than the group that had been infected with SARS-CoV-2 [37]. Krammer et al. also found that the antibody response in participants who had had their first dose of mRNA vaccine was higher in the group that had previously experienced SARS-CoV-2 infection than in the group who had never had it. They also reported that SARS-CoV-2 antibodies formed faster in the group with a history of infection, with antibody titers forming 0–4 days after vaccination. In the group without a history of SARS-CoV-2 infection, formation took an average of 9–12 days after vaccination [38].

This study has limitations. Participants with diabetes, hypertension, and aged above 50 years only accounted for a small number of participants, which can lead to bias in analysis. In addition, variants of SARS-CoV-2, such as alpha, delta, and omicron, can cause bias. COVID-19 incidence and antibody levels after booster vaccination could be higher due to infection by a variant of concern, such as omicron. Ideally, bias can be reduced by assessing the in vitro virus neutralization test from blood samples to assess antibody effectiveness against SARS-CoV-2 variants. However, this study lacked the tools to carry this out. Recall bias in this study may have occurred during retrospective interviews to obtain information of AEFI and infection before or after boosters, even though most of the participants had written data history kept in the government COVID-19 screening application. Time intervals between booster vaccination and participants’ interviews were widely varied, making COVID-19 incidence observation not optimal. This could also have affected the antibody level results among the participants.

## 5. Conclusions

The cumulative incidence of SARS-CoV-2 infection after mRNA-1273 booster vaccination within 5 months reached 18.6%. The incidence per person per month was 3.2%. A history of SARS-CoV-2 infection before booster vaccination was found to be associated with a reduced risk of SARS-CoV-2 infection post-booster vaccination. Age, sex, hypertension, type 2 diabetes mellitus, obesity and AEFI after the booster vaccine were not associated with COVID-19 incidence. Median antibody levels reached 659 BAU/mL in all participants. Antibody levels were found to be associated with infection after booster vaccination and not with age, sex, hypertension, type 2 diabetes mellitus, obesity or AEFI post-booster vaccination.

## Figures and Tables

**Table 1:** Characteristics of research participants.

Variables	n = 300	%	Median, Min–Max
Age			
<50 years	270	90	36 years, 22–59 years
≥50 years	30	10	
Sex			
Male	68	22.7	
Female	232	77.3	
Hypertension			
No	264	88	
Yes	36	12	
Diabetes Mellitus			
No	290	96.7	
Yes	10	3.3	
Obesity (BMI ≥ 25 mg/kg^2^)			
No	149	49.7	BMI 25.02 m^2^/kg, 14.7–49.9 m^2^/kg
Yes	151	50.3	
SARS-CoV-2 infection before booster			
Yes	121	40.3	
No	179	59.7	
SARS-CoV-2 infection within 5 months after booster			
No	244	81.4	
Yes	56	18,6	
History of SARS-CoV-2 infection			
None	61	20.3	
One time	176	58.7	
Two times	60	20	
Three times	3	1	

**Table 2:** Confirmed COVID-19 cases within 5 months of booster vaccination.

Confirmed COVID-19 Cases (People)	Time after Booster Vaccination (Months)
2	1
5	2
11	3
10	4
28	5
Total: 56	

**Table 3:** Bivariate analysis of COVID-19 incidence within 5 months and the variables studied after mRNA-1273 booster vaccination.

Variables	Infection after Booster				Total (n = 300)	RR (95% CI)	*p* Value
	Yes		No				
	N	%	N	%	N		
Age							
≥50 years	3	10	27	90	30	0.51 (0.17–1.53)	0.300
<50 years	53	19.6	217	80.4	270	1.00 (ref)	
Sex							
Female	44	19	188	81	232	1.07 (0.60–1.92)	0.945
Male	12	17.6	56	82.4	68	1.00 (ref)	
Hypertension							
Yes	8	22.2	28	77.8	36	1.22 (0.63–2.37)	0.722
No	48	18.2	216	81.8	264	1.00 (ref)	
Diabetes Mellitus							
Yes	1	10	9	90	10	0.53 (0.08–3.44)	0.694
No	55	19	235	81	290	1.00 (ref)	
Obesity							
Yes	30	19.9	121	80.1	151	1.14 (0.71–1.83)	0.697
No	26	17.4	123	82.6	149	1.00 (ref)	
Infection before booster							
Yes	7	5.8	114	94.2	121	0.21 (0.09–0.45)	<0.001
No	49	27.4	130	72.6	179	1.00 (ref)	
AEFI after booster							
No	1	4.2	23	95.8	24	0.21 (0.03–1.45)	0.060
Yes	55	19.9	221	80.1	276	1.00 (ref)	

**Table 4:** Median antibody titer correlation with variables studied (n = 300).

Variables	Median (BAU/mL)	Min–Max	IQR (BAU/mL)	*p* Value
Age				
<50 years	657	31–5680	819	0.493
≥50 years	757	202–5680	857	
Sex				
Male	622	65–505	959	0.808
Female	661	31–5680	804	
Hypertension				
No	642	31–5680	803	0.080
Yes	799	103–4190	717	
Diabetes Mellitus				
No	657	31–5680	819	0.514
Yes	765	221–2163	898	
Obesity				
No	585	31–5680	791	0.440
Yes	726	66–5680	853	
AEFI after booster				
No	653	31–3585,	1036	0.922
Yes	659	65–5680	823	
Infection after booster				
No	551	31–5680	643	<0.001
Yes	1005	119–5680	1070	

## Data Availability

The data used are available from the corresponding author upon request.
